# Mechanism of ferroptosis in breast cancer and research progress of natural compounds regulating ferroptosis

**DOI:** 10.1111/jcmm.18044

**Published:** 2023-12-22

**Authors:** Anqi Ge, Qi He, Da Zhao, Yuwei Li, Junpeng Chen, Ying Deng, Wang Xiang, Hongqiao Fan, Shiting Wu, Yan Li, Lifang Liu, Yue Wang

**Affiliations:** ^1^ The First Hospital of Hunan University of Chinese Medicine Changsha China; ^2^ People's Hospital of Ningxiang City Ningxiang China; ^3^ Hunan University of Chinese Medicine Changsha China; ^4^ Hunan University of Science and Technology Xiangtan China; ^5^ The First People's Hospital Changde City Changde China

**Keywords:** breast cancer, Ferroptosis, iron ion metabolism, lipid peroxidation, natural compounds

## Abstract

Breast cancer is the most prevalent cancer worldwide and its incidence increases with age, posing a significant threat to women's health globally. Due to the clinical heterogeneity of breast cancer, the majority of patients develop drug resistance and metastasis following treatment. Ferroptosis, a form of programmed cell death dependent on iron, is characterized by the accumulation of lipid peroxides, elevated levels of iron ions and lipid peroxidation. The underlying mechanisms and signalling pathways associated with ferroptosis are intricate and interconnected, involving various proteins and enzymes such as the cystine/glutamate antiporter, glutathione peroxidase 4, ferroptosis inhibitor 1 and dihydroorotate dehydrogenase. Consequently, emerging research suggests that ferroptosis may offer a novel target for breast cancer treatment; however, the mechanisms of ferroptosis in breast cancer urgently require resolution. Additionally, certain natural compounds have been reported to induce ferroptosis, thereby interfering with breast cancer. Therefore, this review not only discusses the molecular mechanisms of multiple signalling pathways that mediate ferroptosis in breast cancer (including metastasis, invasion and proliferation) but also elaborates on the mechanisms by which natural compounds induce ferroptosis in breast cancer. Furthermore, this review summarizes potential compound types that may serve as ferroptosis inducers in future tumour cells, providing lead compounds for the development of ferroptosis‐inducing agents. Last, this review proposes the potential synergy of combining natural compounds with traditional breast cancer drugs in the treatment of breast cancer, thereby suggesting future directions and offering new insights.

## 1 INTRODUCTION

Breast cancer poses a significant health threat to women worldwide. Recent reports indicate that as of 2023, breast cancer has become the most prevalent type of cancer globally, accounting for approximately 12.5% of new cancer cases worldwide.^1^ Women in every country face the risk of developing breast cancer, which can manifest at any age following puberty and is more likely with advancing age.^2^ Internationally, breast cancer causes higher disability‐adjusted life years (DALYs) losses than any other cancer type.^3^ While considerable progress has been achieved in the diagnosis, treatment and prevention of cancer due to advancements in medical technology, the global mortality rate for breast cancer still ranks fifth among all cancer patients.^4^ Currently, the 5‐year survival rate for breast cancer patients is 90%. However, for patients diagnosed with early stage carcinoma in situ, the rate reaches an impressive 99%. Many patients can recover with appropriate treatment, including localized invasive cases, where the 5‐year survival rate can reach 84%. In contrast, patients with metastases at the time of diagnosis face a significantly lower 5‐year survival rate of only 23%.^5^ Complications resulting from recurrence or tumour metastasis are responsible for 90% of breast cancer patient deaths, emphasizing the critical importance of early diagnosis and treatment for patient prognosis.^6^ Multiple factors influence breast cancer, which may be associated with population demographics, lifestyle, genetic factors and environmental influences.^7^ Among different racial groups, Chinese and Japanese women exhibit the highest breast cancer survival rates, whereas black women have the lowest rates.^8^ Age plays a significant role in the incidence and mortality of breast cancer. Women under 25 years of age are rarely diagnosed with this disease, and the median age for breast cancer diagnosis is 61 years.^8^ Family history is a widely recognized risk factor for breast cancer, with approximately 5%–10% of cases attributed to genetic factors. Mutations in the breast cancer 1 gene (BRCA1) and breast cancer 2 gene (BRCA2) are among the most well‐known causes of autosomal dominant breast cancer.^9^ Commonly used prognostic factors for breast cancer include patient age, lymph node status, tumour size and stage, histological type, proliferation markers and hormone receptor status.^10^

Despite significant advances in breast cancer treatment during recent decades, managing metastasis and recurrence remains the foremost challenge.^11^ The development and invasion mechanisms of breast cancer exhibit several notable characteristics,^12^, ^13^ including (1) Tumour cells sustain continuous proliferation signalling, facilitating their continued growth. (2) Tumour cells can counter programmed cell death through various mechanisms to ensure their survival. (3) While breast cancer induces peripheral blood vessel proliferation, the presence of angiogenesis inhibitors does not hinder tumour cells from acquiring essential substances needed for their survival through invasion and metastasis.^14^ (4) Tumour cells have the ability to evade the inhibitory mechanisms of various growth factors. (5) Tumour cells possess infinite replicative potential. (6) Breast cancer cells demonstrate invasiveness and metastatic behaviour, enabling their spread to other body regions through the blood or lymphatic system, fostering the accumulation of gene instability and mutations, and promoting resistance to cell death.^15^ (7) Additionally, breast cancer tumours can boost tissue inflammation by releasing chemical substances, particularly reactive oxygen species, thereby accelerating gene mutation in surrounding tumour cells and hastening the transition from early stage to malignant tumours.^16^ (8) Breast cancer tumour cells undergo metabolic reprogramming known as ‘aerobic glycolysis’.^17^ (9) Breast cancer tumour cells possess mechanisms to evade immune system elimination, enabling their survival and eventual development into solid tumours.^18^ (10) The genome of breast cancer tumour cells exhibits instability, rendering them prone to mutation.^19^ Programmed cell death in breast cancer primarily encompasses ferroptosis, pyroptosis, autophagy, apoptosis and necrosis.^20^

Ferroptosis is an iron‐dependent programmed cell death characterized by abnormal intracellular accumulation of lipid peroxides. It represents a distinct form of cell death separate from apoptosis, necrosis, and autophagy.^21^ Ferroptosis involves intracellular iron deposition, leading to oxidative stress, reactive oxygen species (ROS) generation and lipid peroxide (Lipid‐OOH) accumulation.^22^ Numerous studies have demonstrated the involvement of ferroptosis in breast cancer tumorigenesis, progression, invasion and drug resistance.^23^, ^24^ Inducing ferroptosis in breast cancer cells can effectively inhibit tumour growth, promising the development of novel anti‐breast cancer drugs and overcoming drug resistance.^25^ Furthermore, natural products serve as vital resources for identifying lead compounds in anti‐breast cancer drug discovery.^26^, ^27^ Recent investigations have revealed that several natural medicinal compounds regulate ferroptosis in breast cancer cells, exhibiting anti‐breast cancer effects.^26^, ^27^ With this perspective, this review emphasizes the related targets within the ferroptosis pathway specific to breast cancer and highlights the mechanisms through which natural compounds modulate ferroptosis. Ultimately, this review aims to provide a theoretical foundation for further research, development, and clinical application of ferroptosis, offering a novel direction for drug development and targeted therapy in future breast cancer treatments.

## 2 DISCOVERY PROCESS AND CHARACTERISTICS OF FERROPTOSIS

The exploration of ferroptosis traces back to the 1980s. Notably, the key membrane protein xCT, associated with ferroptosis, was discovered during this period. Subsequently, after more than two decades of development, the classical ferroptosis agonists erastin and RSL3 were identified in 2003 and 2008, respectively, for their ability to induce mortality in BeJLR cells with RAS mutations.^28^ Stockwell et al.^28^ observed that erastin and RSLs did not trigger the formation of apoptotic bodies, autophagosomes or activate the caspase family during cancer cell death. Moreover, inhibiting apoptosis, necrosis or autophagy did not reverse the cell death induced by erastin and RSLs. In 2012, Dixon et al.^29^ proposed ferroptosis as a distinct form of cell death, differing from apoptosis, necrosis and autophagy in terms of morphology, biochemistry and genetics. Morphologically, ferroptosis is characterized by mitochondrial atrophy, reduced or absent mitochondrial cristae, increased mitochondrial membrane density and outer mitochondrial membrane rupture. Biochemically, ferroptosis is primarily characterized by iron ion accumulation, ROS aggregation and lipid peroxidation.

## 3 MAJOR SIGNALLING PATHWAYS REGULATING FERROPTOSIS

**FIGURE 1:**
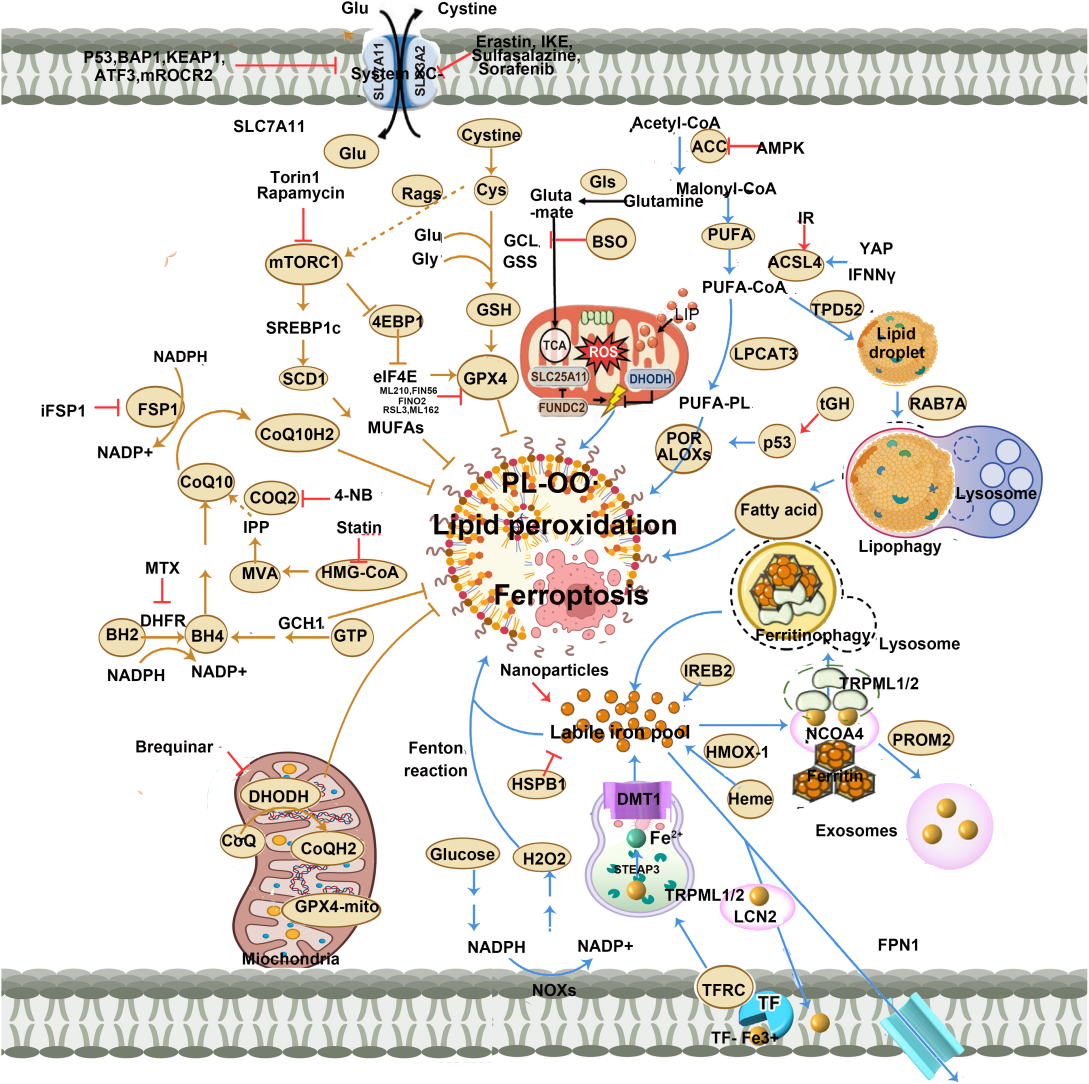
The main mechanism of ferroptosis (The induction and inhibition pathways of ferroptosis help maintain the stability of intracellular lipid peroxides, and they interact and influence each other. For instance, the X‐cys‐system‐GSH‐GPX4, NAD(P)H/FSP1/CoQ10 system, GCH1/BH4/DHFR system, iron metabolism system and mitochondrial respiratory system collectively contribute to the clearance of lipid peroxides, and they are interconnected and function cooperatively. ACC, Acetyl‐CoA carboxylase; ACSL4, acyl‐CoA synthetase long‐chain family member 4; BH2, dihydrobiopterin; BH4, tetrahydrobiopterin; BSO, L‐buthionine sulfoximine; Cys, cysteine; DHODH, dihydroorotate dehydrogenase; FSP1, ferroptosis suppressor protein 1; Glu, glutamate; GSS, Glutathione synthase; HMOX1, heme oxygenase 1; LPCAT3, lysophosphatidylcholine acyltransferase 3; MUFA, monounsaturated fatty acid; MVA, mevalonate pathway; PL, phospholipids; POR, cytochrome p450 oxidoreductase; SCD1, stearoyl coenzyme a desaturase 1; SCD1, stearoyl coenzyme a desaturase 1; TRPML1/2, transient receptor potential channel subfamily).

The main mechanism of ferroptosis is shown in Figure 1.

### 3.1 Inhibition of cystine/glutamate antiporter (system XC^−^) induces ferroptosis

The cell membrane harbours system XC, composed primarily of solute carrier family 3 member 2 (SLC3A2) and solute carrier family 7 member 11 (SLC7A11).^30^ Working in a 1:1 ratio, system XC facilitates the exchange of amino acids, importing extracellular cystine and exporting intracellular glutamate. Once inside the cell, cystine undergoes enzymatic reactions to produce cysteine. The ATP‐dependent cysteine‐glutamate ligase then catalyses the synthesis of γ‐glutamylcysteine using cysteine, which further forms glutathione (GSH) through glutathione synthetase. GSH plays a crucial role in regulating ferroptosis, functioning as a scavenger of free radicals and serving as an vital antioxidant within the human body. Consequently, inhibiting the uptake of cystine by system XC can impede GSH synthesis, leading to the accumulation of superoxide and triggering ferroptosis.

#### 3.1.1 p53 mediates ferroptosis by inhibiting system XC ^−^

p53, a tumour suppressor factor, plays a role in enhancing cell sensitivity to ferroptosis by downregulating the expression of SLC7A11, a key component of system XC^−^, and inhibiting cysteine uptake.^31^ Huang et al.^31^ demonstrated that the ferroptosis inducer, erastin, upregulates p53 expression, subsequently inhibiting system XC^−^ and causing ferroptosis by downregulating SLC7A11. In a similar vein, Le et al. discovered that p53 downregulates SLC7A11, disrupts cystine uptake by system XC^−^, interferes with GSH synthesis and induces ferroptosis.^32^ Current research has shown that several metabolic‐related genes, such as SAT1, FDXR, and GLS2, have been reported as direct targets of p53‐mediated ferroptosis under various conditions, emphasizing the importance of p53 as a modulator of metabolic genes involved in iron death.^33^, ^34^ Furthermore, recent investigations have revealed that p53 not only inhibits nitric oxide‐mediated lipid peroxidation in human colorectal cancer cells by directly binding to dipeptidyl peptidase‐4 (DPP4) but also restricts the ability of iron death by inducing CDKN1A expression in fibrosarcoma cells.^34^ Moreover, current research suggests that lipid peroxidation is not only a key factor in ferroptosis but also that the overall importance of individual p53 target genes or binding proteins in ferroptosis may be cell type‐specific.^35^ Hence, the p53 gene participates in the regulation of ferroptosis through system XC^−^.

#### 3.1.2 Nrf2‐Keap1 induces ferroptosis through system XC

Under normal circumstances, nuclear factor E2‐related factor 2 (Nrf2) forms a complex with Kelch‐like epichlorohydrin‐associated protein 1 (Keap1). However, during oxidative stress, Nrf2 dissociates from Keap1, translocates to the nucleus, initiates the transcription of antioxidant response elements, and activates multiple antioxidant genes, such as SLC7A11, thereby boosting the antioxidant effect of system XC^−^.^36^ Notably, Fan et al.^37^ observed that cancer cells suppressing Nrf2 expression are susceptible to ferroptosis inducers, while cancer cells with elevated Nrf2 expression resist ferroptosis initiation and execution by upregulating system XC^−^. NFE2L2 inhibits oxidative damage in ferroptosis by activating several cell‐protective genes involved in iron metabolism (including SLC40A1, MT1G, HMOX1 and FTH1), glutathione metabolism (including SLC7A11, GCLM and CHAC1) and ROS detoxifying enzymes (including TXNRD1, AKR1C1, AKR1C2, AKR1C3, SESN2, GSTP1 and NQO1).^38^ Functional mutations in NFE2L2 or loss‐of‐function mutations in KEAP1 further compound the complexity of oxidative stress response, which may in turn affect resistance to ferroptosis. The contribution of NFE2L2 to resistance against ferroptosis and the therapeutic potential of NFE2L2 inhibitors (such as Brusatol and Sulforaphane) to enhance ferroptosis need further investigation in preclinical and clinical studies.^39^ Thus, Nrf2 plays a role in modulating ferroptosis through the regulation of system XC^−^.

### 3.2 Inhibition of GPX4 induces ferroptosis

GPX4 is an exclusive enzyme responsible for mitigating lipid peroxidation by utilizing GSH. It has been identified that inhibition of GPX4 activity triggers the onset of phospholipid peroxidation. This pivotal enzyme plays a crucial role in counteracting the peroxidation of cell membrane lipids during the occurrence of ferroptosis. In the context of myocardial infarction, Park et al.^40^ uncovered that suppression of GPX4 led to the accumulation of lipid peroxides, ultimately inducing ferroptosis in cardiomyocytes. Furthermore, solanine was found to hinder GPX4 activity and elevate lipid ROS levels in HCC cells, ultimately resulting in ferroptosis.^41^ Hu et al.,^42^ on the other hand, demonstrated that GPX4 safeguards haematopoietic stem cells by inhibiting lipid peroxidation and impeding the progression of ferroptosis. Consequently, inhibition of GPX4 can facilitate the accumulation of lipid peroxides and promote ferroptosis. Notably, selenocysteine, one of the amino acids in the active centre of GPX4, functions as an essential component in the maintenance of GPX4 activity. It is transported into GPX4 through the transporter‐selenocysteine tRNA, thereby facilitating the scavenging of lipid ROS and inhibiting ferroptosis.^43^ Consequently, the absence of selenium leads to the inhibition of GPX4 activity and, subsequently, induction of ferroptosis.

### 3.3 Iron ‘overload’ induces ferroptosis

**FIGURE 2:**
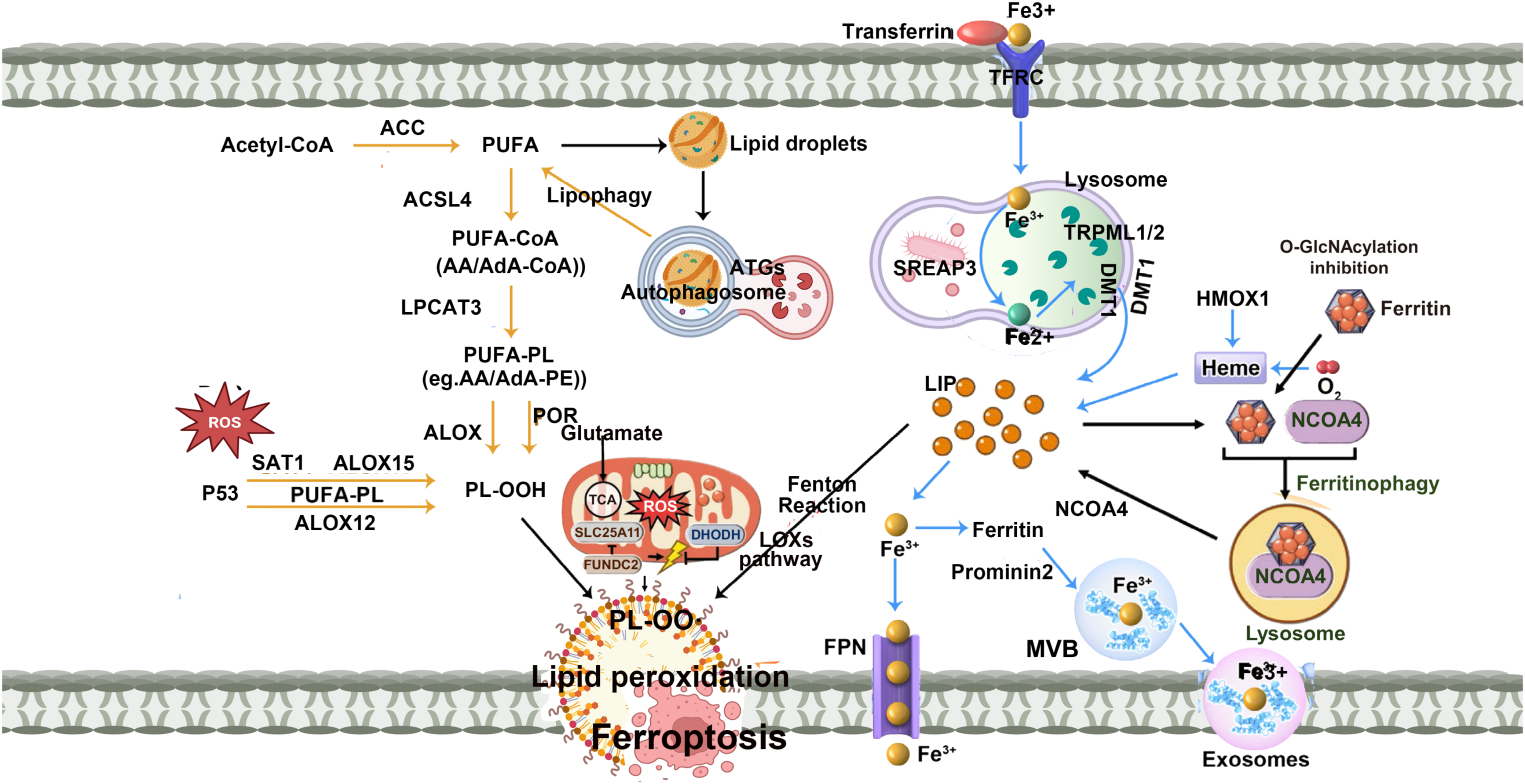
The mechanism of iron ‘overload’ induces ferroptosis (ACC, Acetyl‐CoA carboxylase; ACSL4, acyl‐CoA synthetase longchain family member 4; ALOX, arachidonate lipoxygenases; HMOX1, heme oxygenase 1; LPCAT3, lysophosphatidylcholine acyltransferase 3; PL, phospholipids; POR, cytochrome p450 oxidoreductase; PUFA, polyunsaturated fatty acid; TRPML1/2, transient receptor potential channel subfamily).

Iron accumulation is one of the key factors of ferroptosis, and increased iron intake, reduced efflux, or reduced storage can lead to iron ‘overload’ and thus promote ferroptosis (Figure 2).

#### 3.3.1 Increased iron uptake via transferrin receptor 1 (TFR1)

Research indicates that TFR1 plays a crucial role in facilitating the entry of extracellular Fe^3+^ into the cell's endosomes, where it undergoes reduction to Fe^2+^. This process effectively increases intracellular iron levels, ultimately triggering ferroptosis. In a study conducted by Tang et al.,^44^ they discovered that ubiquitin‐specific protease 7 enhances iron uptake and promotes ferroptosis by activating the p53/TFR1 pathway in the rat heart following ischaemia–reperfusion treatment. Similarly, Jiang et al.^45^ observed that prolonged hyperinsulinemia stimulates hepatic TFR1 expression through the PI3K/IRP2 pathway, resulting in liver iron overload. This condition can significantly contribute to the development of abnormal iron metabolic disorders. Therefore, the upregulation of TFR1 can lead to increased iron intake, resulting in iron ‘overload’ and the promotion of ferroptosis.

#### 3.3.2 Ferritin reduces iron storage through autophagic degradation

Recent studies have significantly contributed to our understanding of the significant impact of autophagy on promoting the occurrence of ferroptosis.^46^ Within cells, ferritin serves as the primary complex responsible for iron storage, playing a crucial role in iron homeostasis. Consisting of two subunits, ferritin light polypeptide 1 (FTL1) and ferritin heavy polypeptide 1 (FTH1), this complex forms an intricate structure.^47^ It is notable that excess Fe^2+^ accumulation within ferritin leads to the formation of an unstable pool of iron. Capitalizing on the power of autophagy, FTH1/FTL1 undergoes degradation, releasing a substantial amount of free Fe^2+^ and elevating intracellular iron levels. Intriguing findings from a study conducted by Yang et al.^48^ uncovered the selective degradation of the core clock protein ARNTL through autophagy. This degradation process suppresses the transcription of Egln2 through ARNTL, ultimately leading to the downregulation of hypoxia‐inducible factor HIF1‐α, which actively induces ferroptosis. Additionally, a separate investigation by Hou et al.^49^ revealed the inhibitory effects on erastin‐induced ferroptosis achieved through the knockout of autophagy‐related genes 5 (Atg5) and 7 (Atg7). Depletion of these genes reduces intracellular ferrous levels and mitigates lipid peroxidation. Notably, the knockdown of nuclear receptor coactivator 4 (NCOA4) hampers ferritin degradation, effectively suppressing the progression of ferroptosis. Conversely, the overexpression of NCOA4 significantly enhances ferritin degradation, thereby promoting ferroptosis. Consequently, autophagy plays a pivotal role in the degradation of ferritin, the reduction of iron reserves and the facilitation of ferroptotic processes.

#### 3.3.3 Limiting iron outflow

The cellular entry of Fe^2+^ can be facilitated through various mechanisms such as divalent metal transporter 1 (DMT1), multivesicular bodies of ferritin and exosomes. Blocking DMT1 or impeding multivesicular bodies and exosomes can restrict iron efflux and elevate intracellular iron levels.^50^ Wang et al.^51^ discovered that oral administration of DMT1 siRNA encapsulated in ginger nanoparticles, as a lipid carrier, effectively reduced iron overload in a mouse model of hereditary hemochromatosis. Furthermore, Turcu et al.^52^ demonstrated that inhibitors targeting DMT1 selectively affect cancer stem cells by inhibiting lysosomal iron transport, resulting in lysosomal iron accumulation, reactive oxygen species generation and ferroptosis induction. Consequently, targeted inhibition of DMT1 can restrain iron efflux, increase intracellular iron levels, and induce ferroptosis.

### 3.4 Promotion of ACSL4/LPCTA3/ALOX15 lipid metabolism pathway induces ferroptosis

The accumulation of iron‐dependent lipid peroxides represents a crucial hallmark of ferroptosis. Recent studies have indicated that the buildup of polyunsaturated fatty acids (PUFAs) serves as a key indicator of ferroptosis, with the intracellular content of PUFAs determining the extent of ferroptotic development.^53^ The process of PUFA accumulation is intricate, and the inhibition of GPX4 leads to the accumulation of PUFAs and ROS, resulting in plasma membrane damage and subsequent ferroptosis. Notably, various fatty acids, including polyunsaturated fatty acids and monounsaturated fatty acids (MUFAs), act as substrates for lipid peroxidation. Among these, PUFA exhibits higher susceptibility to oxidation compared to MUFA.^54^ Therefore, the reduction of MUFA content coupled with an increase in PUFA content actively promotes lipid peroxidation‐induced ferroptosis. Furthermore, three essential enzymes participate in the enzymatic reaction of lipid peroxidation: long‐chain acyl‐CoA synthetase 4 (ACSL4), lysophosphatidylcholine acyltransferase 3 (LPCAT3), and arachidonic acid 15‐lipoxygenase (ALOX15). Among them, ACSL4 plays a vital role in catalysing the esterification of free PUFAs, which are subsequently incorporated into membrane phospholipids with the assistance of LPCAT3. ALOX15, on the other hand, actively contributes to the peroxidation process of membrane phospholipids.^55^ Thus, the ACSL4/LPCAT3/ALOX15 pathway appears to play a crucial role in promoting lipid peroxidation‐induced ferroptosis.

### 3.5 Inhibition of NAD(P)H/FSP1/CoQ10 pathway induces ferroptosis

The NAD(P)H/FSP1/CoQ10 pathway operates as an antioxidant mechanism that runs parallel to GPX4, offering an alternative defence against ferroptosis. Ferroptosis suppressor protein 1 (FSP1), previously known as apoptosis‐inducing factor mitochondrial 2, plays a pivotal role in conferring resistance against ferroptosis.^56^ Located within the plasma membrane, FSP1 functions as an oxidoreductase, facilitating the reduction of ubiquinone (CoQ) to dihydroubiquinone (CoQH2). CoQH2, acting as an antioxidant, effectively scavenges lipophilic free radicals, thereby inhibiting the accumulation of lipid peroxides.^57^ As a result, the suppression of the NAD(P)H/FSP1/CoQ10 pathway's expression can trigger the initiation of ferroptosis.

### 3.6 Blocking the GCH1/BH4/DHFR pathway induces ferroptosis

An additional antioxidant pathway, independent of GPX4, has been identified and is known as the GCH1/BH4/DHFR pathway.^57^ Among these components, guanosine triphosphate cyclohydrolase 1 (GCH1) acts as the crucial rate‐limiting enzyme involved in tetrahydrobiopterin (BH4) synthesis. BH4, in turn, plays a key role in the synthesis of CoQ precursors. Additionally, dihydrofolate reductase (DHFR) assumes a critical role in converting BH2 to BH4 within this pathway. Therefore, the proper functioning of the GCH1/BH4/DHFR pathway promotes CoQ synthesis, effectively restraining the accumulation of lipid peroxides and providing protection against ferroptosis. Intriguingly, research conducted by Soula et al. uncovered that genetic or pharmacological interventions targeting DHFR, in combination with GPX4 inhibition, synergistically induce ferroptosis.^58^ Therefore, the GCH1/BH4/DHFR pathway emerges as an additional parallel antioxidant system alongside GPX4, playing a pivotal role in the defence against ferroptosis.

### 3.7 Inhibition of the dihydroorotate dehydrogenase (DHODH) pathway induces ferroptosis

**FIGURE 3:**
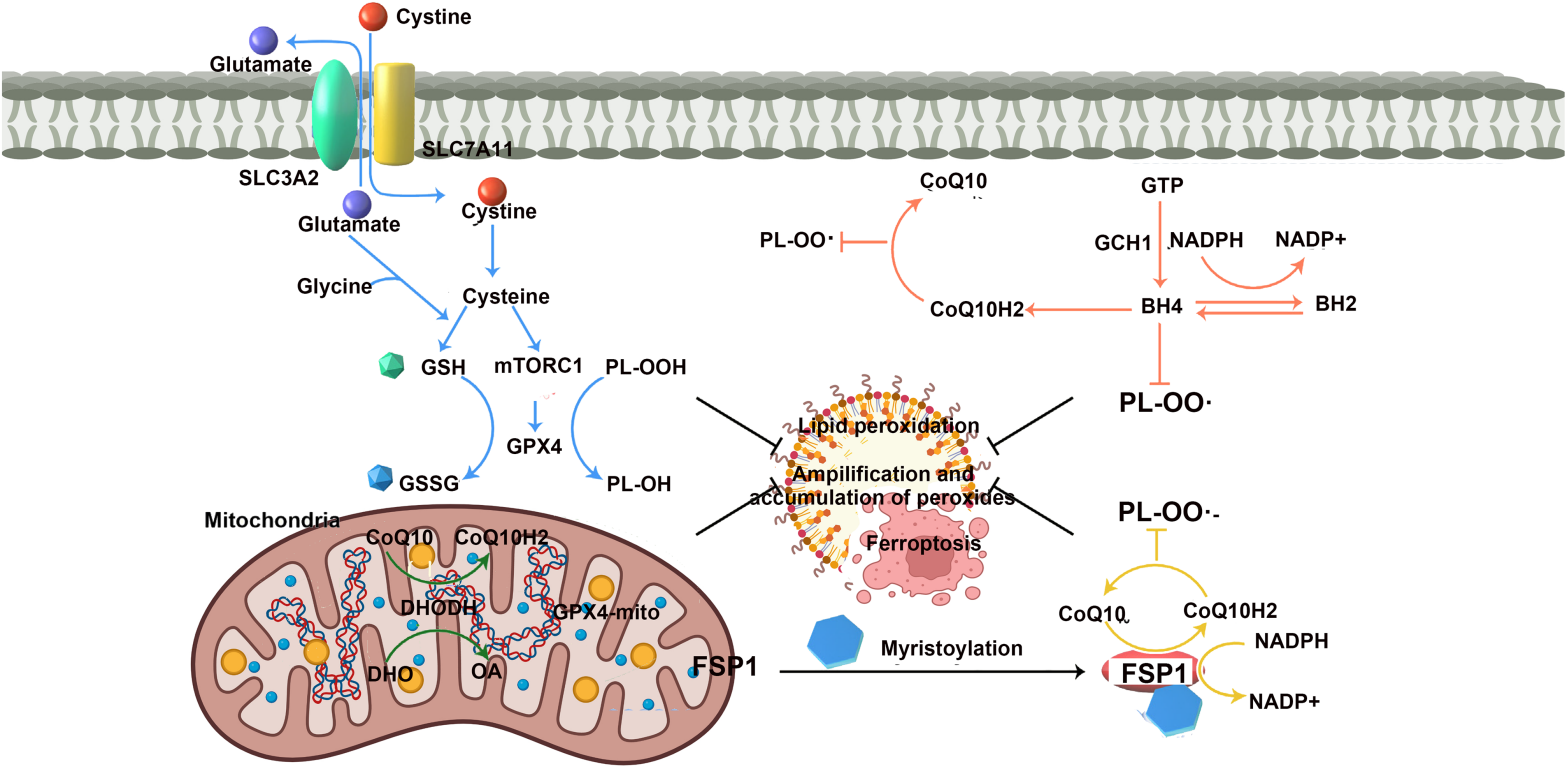
The main pathway mechanism (ALOX, arachidonate lipoxygenases; BH2, dihydrobiopterin; BH4, tetrahydrobiopterin; FSP1, ferroptosis suppressor protein 1; GSH, glutathione; GSSG, oxidized glutathione; PL, phospholipids; TRPML1/2, transient receptor potential channel subfamily).

DHODH, an enzyme that relies on flavin and is located in the inner mitochondrial membrane, plays a critical role in the oxidation of dihydroorotate to orotate. Consequently, it transfers electrons to CoQ, resulting in the reduction of CoQ to CoQH2 and effectively impeding the progression of ferroptosis. It is worth noting that Mao et al.^59^ recently discovered that the addition of the DHODH inhibitor buquinar (BQR) to cell lines exhibiting low GPX4 expression can induce ferroptosis. Conversely, when BQR is introduced to cell lines with high GPX4 expression, it heightens their susceptibility to ferroptosis inducers. Therefore, DHODH emerges as an independent modulator of ferroptosis, operating outside the GPX4 pathway. Targeting the expression of DHODH may thus present a novel strategy for inducing ferroptosis. The main pathway mechanism is shown in Figure 3.

## 4 RESEARCH PROGRESS OF FERROPTOSIS IN TUMOUR DISEASES

Ferroptosis plays a dual role in disease progression, acting both as an inhibitor and a promoter. In recent years, conventional therapies have proven ineffective against treatment‐resistant tumour cells in cancer treatment. However, emerging studies have shown that various tumour cells are highly sensitive to ferroptosis inducers, which can selectively trigger the death of tumour stem cells, enhance the sensitivity of tumour cells to chemotherapy drugs, and eliminate cancer cells. Consequently, the induction of ferroptosis exhibits potential as an anticancer approach. Moreover, mounting evidence suggests a connection between ferroptosis and neoplastic diseases.^60^, ^61^, ^62^, ^63^ For instance, in liver cancer, Wu et al.^64^ identified Sorafenib, a ferroptosis inducer, as inhibiting system XC activity to induce ferroptosis, impede tumour angiogenesis, suppress the proliferation of liver cancer cells, and reduce the recurrence rate of liver cancer post‐surgery. Additionally, Sun et al.^65^ revealed that Restin Thiooxidase 1 heightened the sensitivity of hepatoma cells to Sorafenib‐induced ferroptosis by inhibiting Nrf2 activation. Furthermore, studies have demonstrated that the ferroptosis inducer dihydroartemisinin reduced GSH content, downregulated GPX4 protein levels, increased ROS levels, induced ferroptosis, and inhibited liver cancer cell proliferation.^66^ Hence, interventions targeting drug‐induced ferroptosis offer a promising avenue for liver cancer treatment. Similarly, in pancreatic cancer, Badgley et al.^67^ discovered that pancreatic cancer cells are highly reliant on cysteine concentrations. These cells transport cysteine via system XC, synthesize GSH and coenzyme A, and thereby resist ferroptosis. Under a transmission electron microscope, cysteine‐deprived cancer cells exhibited distinct pathological features associated with ferroptosis, including smaller mitochondria, increased double‐membrane structure density, and the absence of mitochondrial cristae. This compelling evidence affirms that the induction of ferroptosis inhibits the growth of pancreatic cancer cells. In a related study, Du et al.^68^ demonstrated the collaborative induction of ferroptosis by dihydroartemisinin and cisplatin, which elevated cellular levels of ROS and ultimately hindered pancreatic cancer cell proliferation. Hence, the exploration of ferroptosis induction as a therapeutic strategy for pancreatic cancer shows immense potential. In the context of lung cancer, studies have demonstrated a positive association between higher serum iron concentrations, ferritin levels, and both the incidence and mortality rates of the disease.^69^ For instance, Guo et al.^70^ revealed that the ferroptosis‐inducing agent erastin exerts its effect through GSH depletion and GPX4 inactivation, thereby synergistically inhibiting the proliferation of lung cancer cells in combination with cisplatin. Additionally, Wang et al.^71^ discovered that the stem cell factor SOX2 upregulates the expression of SLC7A11, thereby enhancing the resistance of lung cancer cells to ferroptosis. Interestingly, following SOX2 knockdown, SLC7A11 expression was downregulated, resulting in a significant decrease in cellular GSH levels and increased sensitivity to ferroptosis. Furthermore, Hu et al.^72^ identified a pronounced upregulation of SLC7A11 in KARS mutants, such as lung adenocarcinoma patients, which exhibited a positive correlation with tumour progression. Notably, the knockout of the SLC7A11 gene or its inhibition effectively impeded cystine uptake and GSH synthesis, leading to significant inhibition of KARS mutant tumour cell growth. Consequently, the utilization of ferroptosis‐inducing agents in lung cancer treatment demonstrates considerable promise in reducing the recurrence rate and mortality among affected individuals.

## 5 CORRELATION BETWEEN FERROPTOSIS AND BREAST CANCER

### 5.1 The tumour suppressor effect of ferroptosis

The unique metabolic characteristics of cancer cells, including abnormal amino acid metabolism and their dependency on specific amino acids, provide potential opportunities for targeted cancer treatments. In their investigation, Chen et al.^73^ made a notable discovery in triple‐negative breast cancer (TNBC) cells, revealing that the deficiency of cysteine (Cys) in these cells is highly lethal. However, the administration of deferoxamine (DFO) and ferrostatin‐1 effectively blocked the cell death induced by Cys deficiency. This compelling evidence suggests that Cys deficiency can indeed induce ferroptosis in TNBC cells. Furthermore, Yu et al.^74^ conducted a separate study in breast cancer ZR‐75‐1 cells, which unveiled an intriguing finding. The administration of sulfasalazine (SAS), a well‐known compound used for various medical conditions, resulted in the inhibition of xCT protein and GPX4 protein. These proteins play crucial roles in the system XC^−^. Additionally, SAS enhanced the expression of divalent metal ion transporter 1 (DMT1). As a result, this unique combination substantially accumulated reactive oxygen species within the breast cancer cells, ultimately triggering ferroptosis. These findings underscore the potential significance of targeting amino acid metabolism to induce ferroptosis in breast cancer cells, thereby offering a promising therapeutic strategy.

### 5.2 Participate in synergistic anticancer effect

Ma et al.^75^ conducted a study demonstrating the synergistic induction of cell death in breast cancer cell lines MDA‐MB‐231, MCF‐7, ZR‐75 and SKBr3 by combining siramesine and lapatinib. The cell death mechanism was associated with increased levels of lipid‐reactive oxygen species (Lip‐ROS) and FeCl3 within the cells. Furthermore, the researchers discovered that ferrostatin‐1 and DFO could reverse the cell death induced by siramesine and lapatinib. Interestingly, lapatinib alone or in combination with siramesine reduced the expression of iron transporters, resulting in decreased iron export to the extracellular space. Simultaneously, it elevated transferrin expression, facilitating iron transport into the cells, increasing cellular iron content and catalysing iron‐dependent Lip‐ROS production. Overexpression of ferroportin or silencing of transferrin expression reduced intracellular Lip‐ROS production and inhibited cell death. These findings suggest that the synergistic anticancer effect of siramesine and lapatinib occurs through the ferroptosis pathway.

### 5.3 Enhance radiosensitivity

The utilization of holo‐lactoferrin (Holo‐Lf), an iron‐saturated form of lactoferrin, has demonstrated potential in enhancing erastin‐induced ferroptosis in MDA‐MB‐231 cells as well as augmenting cell sensitivity to radiotherapy. In a study conducted by Zhang et al.,^76^ it was revealed that Holo‐Lf not only inhibited the viability of MDAMB‐231 cells but also further suppressed cell viability when combined with erastin. This combination resulted in an increased propidium iodide (PI) fluorescence value within the cells and elevated production of Lip‐ROS. Conversely, iron‐deficient lactoferrin (Apo‐Lf) significantly reduced the PI fluorescence value in MDA‐MB‐231 cells and diminished the generation of Lip‐ROS, indicating distinct impacts on the ferroptosis process. Furthermore, the study identified a significant down‐regulation of GPX4 expression induced by Holo‐Lf in MDA‐MB‐231 and MCF‐7 cells. In contrast, Apo‐Lf displayed a notable up‐regulation of GPX4 expression in these cell types. Notably, when Holo‐Lf was combined with radiotherapy at a dose of 4 Gy in the treatment of MDA‐MB‐231 cells, it substantially increased the production of Lip‐ROS. The observation suggests that radiotherapy has the potential to promote ferroptosis in cells and enhance radiosensitivity. These findings underscore the potential of Holo‐Lf in combination with radiotherapy as a strategy to induce ferroptosis, thereby enhancing the efficacy of radiotherapy in cancer treatment.

### 5.4 Inhibit distant metastasis

Distant metastasis and recurrence are the leading causes of breast cancer‐related mortality,^77^ with brain metastasis representing an independent risk factor for HER‐2‐positive breast cancer. Although HER‐2‐targeted therapy has shown promise in extending the survival of HER‐2‐positive breast cancer patients, a significant challenge remains. Approximately 50% of patients with brain metastases fail to achieve a cure due to chemotherapeutic drug resistance and limited drug penetration across the blood–brain barrier. Neratinib, a tyrosine kinase inhibitor, plays a crucial role in treating patients with HER‐2‐positive breast cancer.^78^ In their study, Nagpal et al. transplanted the HER‐2‐highly expressing TBCP‐1 cell line into nude mice and observed higher incidences of spontaneous brain metastasis, as well as metastasis to other organs. Furthermore, they demonstrated that neratinib effectively induced cell death in vitro.^79^ Remarkably, the authors discovered that neratinib‐induced cell death occurred via a non‐apoptotic mechanism, which could be reversed by the ferroptosis inhibitor liproxstatin‐1, but not by the apoptosis inhibitor Q‐VD. These findings indicate that neratinib induces the death of TBCP‐1 cells through the ferroptosis pathway. In addition, neratinib significantly inhibited tumour growth and metastasis to the liver, lung, and brain in nude mice, strengthening the correlation between neratinib and the activation of the ferroptosis pathway.^79^

### 5.5 Combining exosomes to enhance anticancer effect

Exosomes, known for their targeting abilities and biocompatibility, provide unique advantages in cancer therapy by enhancing the targeted delivery of anticancer drugs to tumours. In a study carried out by Yu et al.,^80^ a novel approach utilizing erastin‐loaded exosomes (erastin@FA‐exo) with folic acid (FA) labelling was investigated. This approach was specifically designed to target TNBC cells that overexpress the FA receptor. Comparative analysis with free erastin revealed that the erastin@FA‐exo formulation exerted a notable inhibitory effect on the proliferation and migration of MDA‐MB‐231 cells. This effect was attributed to its ability to target and modulate GSH, resulting in the down‐regulation of GPX4 expression. Consequently, Lip‐ROS accumulated within the cells, ultimately triggering ferroptosis.

### 5.6 Other

Small molecule‐induced ferroptosis has emerged as a promising strategy for inhibiting tumour growth and enhancing sensitivity to chemotherapeutic drugs. The xCT protein serves as a functional subunit of systemXc, responsible for the conversion of intracellular glutamate and extracellular Cys, playing a critical role in the survival of TNBC. Abnormally high expression of the mucin 1 C‐terminal subunit (MUC1‐C) transmembrane oncoprotein has been observed in TNBC cells. MUC1‐C interacts with xCT to maintain the redox balance of GSH. In their study, Hasegawa et al. demonstrated that silencing MUC1‐C expression in TNBC cells resulted in the downregulation of xCT expression, imbalanced GSH levels and induction of cell death mediated by reactive oxygen species. Importantly, the ferroptosis inhibitor, ferrostatin‐1, was able to reverse the cell death caused by silencing MUC1‐C expression.^81^ Furthermore, the study revealed that TNBC cells with silenced MUC1‐C expression exhibited increased sensitivity to erastin‐induced cell death when exposed to doxorubicin, highlighting the anti‐ferroptotic effect of MUC1‐C. These findings suggest that targeting the MUC1‐C/xCT pathway may hold promise as a potential therapeutic approach to induce cell death in TNBC cells.

## 6 SIGNALLING PATHWAYS REGULATED BY FERROPTOSIS IN BREAST CANCER

### 6.1 Ferroptosis is involved in the epithelial–mesenchymal transition (EMT) of breast cancer

Recent studies have uncovered a significant association between ferroptosis and the epithelial‐to‐mesenchymal transition (EMT) in breast cancer (BC) cells, suggesting the involvement of kinase inhibitor‐induced changes in lipid metabolism.^82^ Activation of the PI3K/AKT/mTOR pathway constitutes a major resistance mechanism against small molecule inhibitors. Altered lipid metabolism contributes to acquired resistance to HER2‐targeted therapy by bypassing upstream growth factor receptor inhibition. Notably, the PI3K/AKT/mTOR pathway plays a crucial role in regulating FA synthesis and FA oxidation.^83^, ^84^ Encouragingly, recent investigations have demonstrated that rapamycin‐mediated mTOR inhibition leads to a significant increase (60%) in β‐oxidation, while its inhibitor AMPK restores energy balance by augmenting FA oxidative signalling and suppressing FA synthesis.^85^, ^86^

Additionally, studies have unveiled that breast cancer cells exhibiting a mesenchymal phenotype display remarkable susceptibility to ferroptosis and exhibit positive responses to therapeutic interventions.^87^, ^88^, ^89^ GPX4, a pivotal player conferring treatment resistance, heavily relies on breast cancer cells in a mesenchymal state. Consequently, inhibiting GPX4 renders these tumour cells highly susceptible to ferroptosis.^90^, ^91^ Notably, the discoidin domain receptor tyrosine kinase 2 (DDR2), induced by EMT regulators TWIST and SNAIL, exhibits elevated expression levels in mesenchymal breast cancer cells. Interestingly, treatment with erastin results in enhanced DDR2 upregulation and phosphorylation. Silencing DDR2 in recurrent BC cells with mesenchymal features reduces YAP/TAZ expression and clonal proliferation by inducing ferroptosis.^92^ Administration of BET and proteasome inhibitors has demonstrated efficacy against mesenchymal subtypes of TNBC and has been found to trigger ferroptosis. Further investigations have revealed that these cells display a distinct susceptibility to ferroptosis inducers, exhibiting enrichment of ferroptosis gene signatures and differential expression of key proteins that elevate labile iron levels while reducing GSH concentrations.^93^

In summary, the therapeutic potential of ferroptosis has been demonstrated against mesenchymal breast cancer cells, characterized by their EMT capabilities and resistance to conventional therapies. Kinase inhibitors targeting HER2/4 or CDK4/6 within the PI3K/AKT/mTOR pathway have been utilized in breast cancer treatment and shown to regulate lipid metabolism. Combining these inhibitors with ferroptosis inducers could present a promising therapeutic strategy for breast cancer management.

### 6.2 Ferroptosis involved in the development, drug resistance and invasion of TNBC

Current research on ferroptosis in breast cancer primarily focuses on TNBC due to its high susceptibility to relapse and drug resistance. Inhibition of the xCT ferroptosis signalling pathway using erastin and SAS has been shown to enhance ferroptosis, leading to increased accumulation of ROS in MDA‐MB‐231 cells and 4T1 mouse TNBC cells.^80^, ^94^, ^95^ Remarkably, cystine starvation induces mitochondrial fragmentation, dysfunction and ROS production in TNBC through upregulation of ATF4 and CHAC1. Knockdown of CHAC1 rescues GSH reduction and inhibits ferroptosis triggered by cystine starvation.^73^ In addition, targeting GPX4, a ferroptosis inhibitor, effectively suppresses TNBC growth with minimal side effects.^96^, ^97^ PROMININ2 has been found to promote ferroptosis resistance by facilitating iron efflux from cells, indicating the development of resistance to ferroptosis in TNBC.^50^ Furthermore, Brown et al.^98^ have discovered that TNBC cells survive by activating the PVRL4/α6β4/SRC signalling axis, which maintains GPX4 expression and protects against lipid peroxidation. In a mouse model of ferroptosis, Doll et al.^90^ demonstrated that pharmacological targeting of ACSL4 enhances TNBC cell death, providing a potential therapeutic strategy to promote ferroptosis. Moreover, the combination of lysosomal disruptors, such as selamectin and lapatinib, induces autophagy and ferroptosis in TNBC cells.^75^, ^99^

Investigation of the relationship between autophagy and ferroptosis has revealed that BRD4 knockdown or JQ1 application induces ferroptosis in MDA‐MB‐231 and Hs578T cells, which is further enhanced when combined with RSL3, sorafenib and erastin. Additional studies have shown that JQ1 either stimulates ferritin phagocytosis or represses the transcriptional expression of GPX4, SLC7A11 and SLC3A2, providing new insights into TNBC treatment with JQ1.^100^, ^101^ Furthermore, ferroptosis has demonstrated its ability to eliminate breast cancer stem cells (BCSCs), which are resistant to conventional therapies and associated with metastasis and recurrence. Salinomycin‐induced ferroptosis exhibits potent and selective activity against BCSCs by promoting iron accumulation and sequestration in lysosomes.^89^, ^102^, ^103^, ^104^ These findings underscore the critical role of ferroptosis in the development, metastasis and invasion of breast cancer.

### 6.3 Ferroptosis mediated by GSH metabolic pathway involved in breast cancer

The levels of GSH are significantly higher in breast cancer tissue compared to non‐cancerous tissue and elevated GSH has been associated with an increased risk of breast cancer.^104^, ^105^, ^106^ Intriguingly, GSH levels are found to increase in sentinel lymph nodes exhibiting micrometastases and macrometastases, while being reduced in sentinel lymph nodes of patients with stage I‐III breast cancer who have undergone surgery.^107^, ^108^ GSH has been observed to be higher in TNBC compared to the luminal subtype, possibly due to the oestrogen‐mediated reduction of transferrin receptor (TFR) expression in breast cancer.^95^, ^109^ Moreover, the excess of GSH in breast cancer leads to treatment resistance by attenuating apoptosis mediated by the ROS‐abundant environment induced by anticancer drugs such as cisplatin and paclitaxel.^97^ Although these anticancer drugs can induce ferroptosis, they are detoxified by GSH in breast cancer. However, when benzothiazoles are used to inhibit GSH production, nearly 99% of breast cancer cells are killed by cisplatin.^110^, ^111^, ^112^ Downregulation of oestrogen‐related receptor α expression has been found to enhance glutathione levels and increase breast cancer resistance to lapatinib, another ferroptosis inducer. This intervention effectively counteracts ROS production and reverses the sensitivity to lapatinib.^113^ Furthermore, in the context of radiation therapy, which stimulates ROS generation and ferroptosis,^114^ reduction of GSH has been observed to alleviate radiation resistance in breast cancer cells through the hypoxia‐inducible factor‐1 (HIF‐1)‐mediated metabolic reprogramming pathway.^115^ Depletion of GSH using buthionine sulfoximine has been shown to restore radiosensitivity in breast cancer.^116^ Understanding the intricacies of GSH metabolism is crucial for comprehending both ferroptosis and the efficacy of conventional anti‐breast cancer treatments. The glutathione pathway demonstrates a strong association with breast cancer progression and therapy resistance, highlighting its significance in therapeutic strategies.

### 6.4 Ferroptosis mediated by iron metabolic pathway involved in breast cancer

Elevated dietary iron intake has been identified as a contributor to oxidative stress and DNA damage, thereby increasing the risk and recurrence of breast cancer, particularly in BRCA1 carriers and ER^−^/PR^−^ subtypes during chemotherapy.^117^, ^118^, ^119^, ^120^ Various molecules involved in iron homeostasis and tumorigenesis have been detected in the tissue and gut metagenomes of breast cancer patients, including signal activator of transcription 5 (STAT5), STAT3, bone morphogenetic protein 6 (BMP6), cluster of differentiation 74 (CD74), TFR, inhibin alpha (INHA), iron exporter ferroportin (FPN), ferritin heavy chain (FHC) and ferritin light chain (FTL).^121^ In summary, iron overload has been implicated in increasing breast cancer risk and contributing to treatment failure. However, there is still controversy surrounding this view as iron overload can also act as an activator of ferroptosis. Therefore, further exploration is necessary to elucidate the role of iron in breast cancer.

In oestrogen receptor‐positive (ER^+^) breast cancer, both oestrogen and iron exert influence on cancer proliferation.^122^ The combined stimulation of oestrogen and iron has been shown to have synergistic effects on breast cancer risk and enhanced proliferation of MCF‐7 cells, as evidenced by increased expression of Ki67.^123^, ^124^ Moreover, elevated iron concentrations have been observed during the metastatic phase of breast cancer. MDA‐MB‐231 cells with higher intracellular iron levels displayed enhanced expression of mesenchymal markers and activation of tumour necrosis factor α‐induced NF‐κB and transforming growth factor β signalling.^125^ Additionally, iron contributes to chemoresistance through the local IL‐6 paracrine loop in breast cancer cells. Targeting iron disrupts the interaction between breast cancer cells and tumour‐associated macrophages, blocking the IL‐6 signalling pathway, and ultimately overcoming chemoresistance.^126^ In summary, iron emerges as a crucial factor promoting both ferroptosis and the progression of breast cancer. Additional research is needed to gain a comprehensive understanding of the intricate relationship between iron and breast cancer.

### 6.5 Ferroptosis mediated by NRF2 signalling pathway involved in breast cancer

NRF2 is a stress‐inducible transcription factor that is normally kept at low levels by KEAP1. However, during oxidative stress, NRF2 is released from KEAP1 and translocates into the nucleus, activating genes involved in oxidative stress response, mitochondrial function, oxygen consumption, and cellular adenosine triphosphate content.^127^ Prolonged activation of NRF2 in cancer cells leads to therapy resistance, aggressive tumour behaviour, and even “NRF2 addiction”.^128^, ^129^ NRF2 targets various proteins and enzymes involved in preventing lipid peroxidation, thereby inhibiting ferroptosis. Some of these targets related to iron metabolism include ferritin, ferroportin (FPN), quinone oxidoreductase 1, and heme oxygenase 1.^130^, ^131^ Blocking NRF2 makes breast cancer cells susceptible to differentiation and cytotoxicity induced by ROS.^132^ Additionally, NRF2 mediates GSH synthesis through XCT and GCL in breast cancer by binding to antioxidant response elements.^133^, ^134^ NRF2 also plays a role in the detoxification of GST and GPX through nuclear translocation and subsequent transcription.^135^, ^136^ However, the regulation of iron by NRF2 in breast cancer is still poorly understood, despite its significant impact on iron metabolism. Moreover, NRF2 promotes the malignancy of breast cancer through its interaction with HIF‐1α. Downregulating NRF2 reduces the proliferation of MCF‐7 and MDA‐MB‐231 cells, which is then counteracted by HIF‐1α, a process partially regulated by the competing endogenous factor miR‐181c.^137^, ^138^ NRF2 further facilitates breast cancer cell migration by upregulating the HIF‐1α/NOTCH1 axis.^139^ Conversely, the ROS‐NRF2‐ARE pathway upregulates the NOTCH1 receptor in breast cancer, resulting in enhanced proliferation.^140^ Other factors, such as SOD2, SOD3, PRX3, NQO1, UDP‐glucuronosyltransferase, sulfotransferase, and 8‐oxoguanine, are also under the control of NRF2 in breast cancer.^141^, ^142^ The antioxidant effects mediated by NRF2 contribute to drug resistance in breast cancer. P53 missense mutants collaborate with NRF2 to activate proteasome gene transcription in breast cancer, leading to resistance against the proteasome inhibitor carfilzomib and lipid nanocapsules.^143^, ^144^ Among the drug transporters assessed, NRF2 upregulates P‐gp and breast cancer resistance protein (BCRP), resulting in decreased drug import.^145^, ^146^ However, there is a case where NRF2 reverses drug resistance in breast cancer by reducing RON expression. RON, a tyrosine kinase receptor, is abnormally expressed in breast cancer and linked to tamoxifen resistance, suggesting a dual role for NRF2 in this context.

### 6.6 Ferroptosis mediated by ALOXs pathway involved in breast cancer

ALOXs have been shown to play a crucial role in triggering ferroptosis by oxidizing PUFA‐PE.^55^ These enzymes are significant contributors to the ferroptotic process by generating lipid hydroperoxides, and their inhibition has been demonstrated to prevent ferroptosis induced by GPX4 inhibition.^91^, ^147^ When ROS levels surpass the neutralizing capacity of GSH, ALOXs facilitate lipid peroxidation, ultimately leading to ferroptosis. Inhibiting ALOXs has exhibited promise in hindering bone metastasis in breast cancer by blocking AA.^148^ Intriguingly, research has unveiled the complex involvement of ALOXs in breast cancer. While ALOX activity is necessary for the activation of FAK and the formation of STAT5‐DNA complexes, promoting the migration of MDA‐MB‐231 cells,^149^, ^150^ the metabolite docosahexaenoic acid, catalysed by ALOXs, holds potential as an anti‐breast cancer agent.^151^ This complexity suggests that the role of ALOXs in breast cancer may stem, at least partly, from their induction of ferroptosis. ALOX5 has been implicated in the pathophysiological process of breast cancer and is associated with increased breast cancer risk.^152^ In fact, serum ALOX5 levels were found to be nearly twofold higher in breast cancer patients compared to controls.^153^ While a positive correlation between ALOX expression and TNM stage was observed in clinical breast cancer cases, no significant association was found with ER, PR, and HER2.^154^ Another study found that ALOX5 activation was correlated with HER2 expression and mediated breast cancer growth and migration,^155^ indicating the need for further investigation into the relationship between ALOX5 and HER2. Furthermore, knockdown of ALOX5 augmented cell death in MDA‐MB‐231 cells in vitro, suggesting its key role in reducing tumour proliferation.^153^ ALOX5 can be upregulated by AA, and an ALOX5 inhibitor decreased migration in TNBC cells but not in MCF‐7 cells, aligning with the aforementioned epidemiological evidence.^156^ Furthermore, ALOX5 expression was reduced in tumour‐associated macrophages (TAMs) generated by co‐culturing human macrophages with MCF‐7 breast cancer cells, resulting in cell death. The decrease in ALOX5 in TAMs was mechanistically linked to attenuated T cell recruitment.^157^ Genetic or pharmacological inhibition of ALOX5 also eliminates the pro‐metastatic activity of neutrophils, thereby reducing breast cancer metastasis.^158^ Regarding ALOX12, an increased risk of breast cancer was observed in individuals with the ALOX12 polymorphism in their polyunsaturated fat intake; however, no increase in mortality was observed.^158^ Further investigation revealed an approximately twofold higher relative risk of breast cancer associated with ALOX12 polymorphisms compared to individuals with heterozygous variants.^159^ Serum ALOX12 levels were also significantly elevated in patients with lymph node involvement and decreased in over 75% of patients after chemotherapy.^160^ In addition to ALOX5 and ALOX12, ALOX15 has been identified to play a critical role in ferroptosis. Studies in British Columbia revealed a low prevalence of ALOX15 staining in metastatic patient specimens.^161^ Furthermore, ALOX15 expression in human sentinel lymph node metastases showed an inverse correlation with metastasis‐free survival, and knockdown of ALOX15 antagonized the formation of lymph node metastases in xenograft tumours.^162^ In in vitro assays, the major ALOX15 metabolite, 13‐S‐hydroxyoctadecadienoic acid, demonstrated dose‐ and time‐dependent inhibition of breast cancer growth.^163^, ^164^ Additionally, simultaneous targeting of ALOX15 enhances the anticancer potential of 1,2,3‐triazoles by augmenting oxidative stress.^165^ Similarly, ALOX15 is transcriptionally downregulated in DOX‐resistant breast cancer cells, and its overexpression in resistant cells leads to resensitization to DOX. Collectively, ALOXs play crucial roles in oxygen metabolism and ferroptosis in breast cancer. Understanding the involvement of ALOXs in promoting oxidative stress in breast cancer cells may pave the way for novel ferroptosis‐based therapies.

### 6.7 Ferroptosis mediated by NAD(P)H/FSP1/CoQ10 pathway involved in breast cancer

Recent studies have revealed that FSP1 operates as an alternative anti‐ferroptosis mechanism complementing the Cyst(e)ine‐GSH‐GPX4 axis. By utilizing NAD(P)H, FSP1 reduces CoQ10, which in turn reduces lipid free radicals, effectively inhibiting lipid peroxidation. Coenzyme Q, acting as an anti‐oxidant and lipophilic free radical trapping agent, prevents the propagation of lipid peroxides and exerts an inhibitory effect on ferroptosis. The expression of FSP1 positively correlates with ferroptosis resistance in numerous cancer cell lines, as FSP1 prevents ferroptosis induced by erlastine, sorafenib and RSL3.^56^, ^166^ Pharmacologically targeting FSP1 synergizes strongly with GPX4 inhibitors, leading to enhanced cancer ferroptosis. Interestingly, CoQ10 has been implicated in breast cancer risk, with several studies revealing significant associations.^167^, ^168^ Furthermore, CoQ10 levels are significantly reduced in breast cancer tissue compared to surrounding normal tissue.^169^ A study found negative and significant correlations between CoQ10 and the fold changes of AMPK, VEGF‐A and VEGFR2.^170^ However, two studies reported an increase in CoQ10 levels in breast cancer patients.^171^, ^172^ In a clinical study involving end‐stage breast cancer patients receiving CoQ10 supplementation, the median predicted survival was longer than average, with minimal adverse effects.^171^ Additionally, daily CoQ10 supplementation in breast cancer patients with lymph node invasion resulted in significant tumour remission.^171^, ^173^ Co‐administration of CoQ10 with tamoxifen in breast cancer patients increased anti‐oxidant activity while decreasing levels of angiogenic markers and lipids.^174^ Furthermore, reduced CoQ10 in breast cancer cells promotes cell death by inhibiting the pentose phosphate pathway.^175^ The main mechanism underlying the effects of CoQ10 on breast cancer pathophysiology appears to be linked to its regulation of oxidative damage.

## 7 NATURAL COMPOUNDS TARGETING FERROPTOSIS IN BREAST CANCER

**FIGURE 4:**
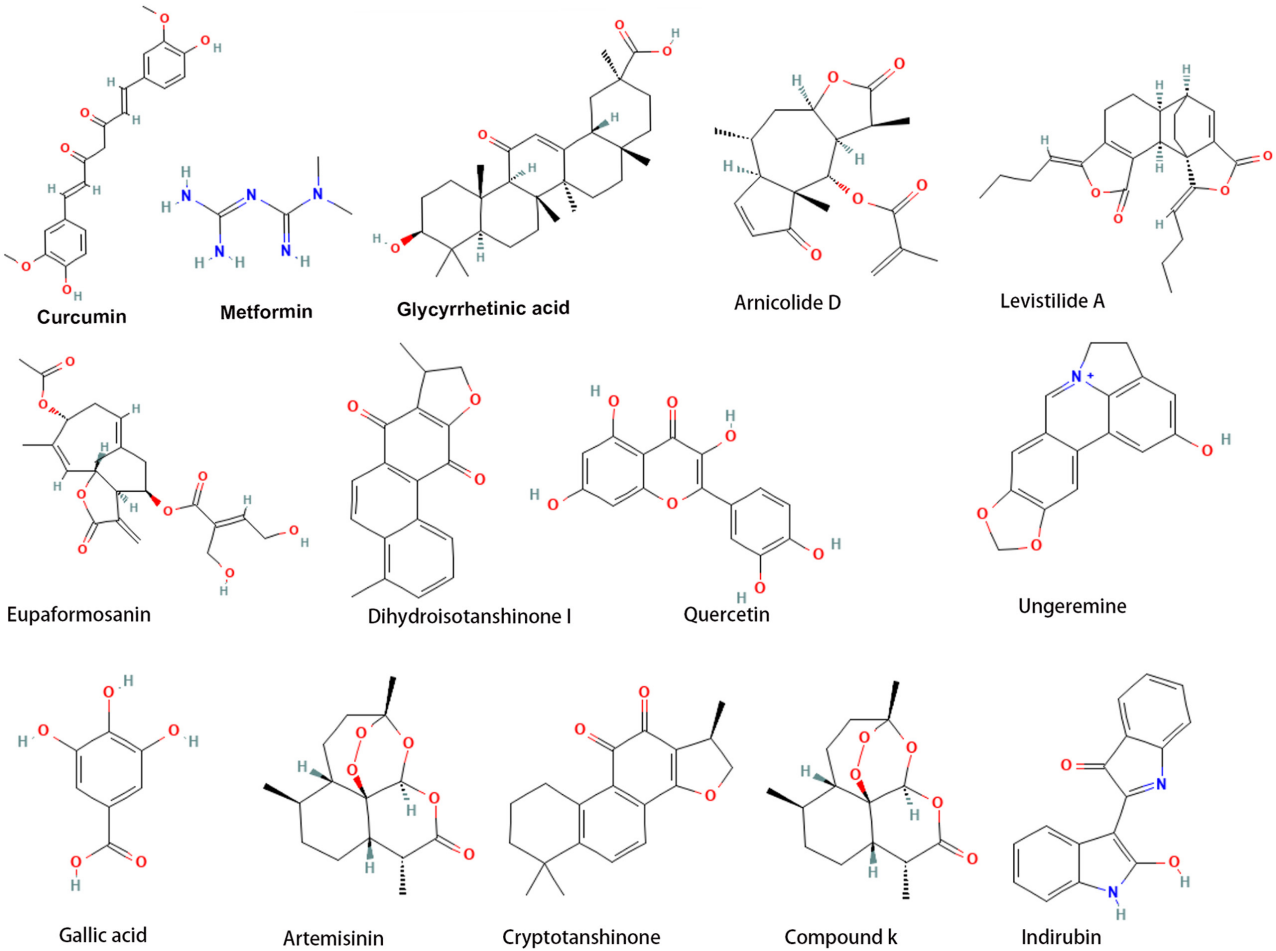
The structures of natural compounds.

The structures of natural compounds are shown in Figure 4. The mechanisms of natural compounds targeting ferroptosis in breast cancer are summarized in Table 1.

**TABLE 1:** The mechanisms of natural compounds targeting ferroptosis in breast cancer.

Natural plant active ingredients	Model/Disease	Species	Effects	References
Curcumin	MCF‐7 cell line	*Homo sapiens*	Upregulate HO‐1 and downregulate GPX4 to mediate ferroptosis in breast cancer cells	^183^
Curcumin	MDA‐MB‐453 cell line and MCF‐7 cell line	*Homo sapiens*	Enhances lipid ROS levels, lipid peroxidation end product MDA accumulation, and intracellular Fe^2+^ levels, thereby promoting SLC1A5‐mediated ferroptosis in breast cancer cells	^184^
Metformin	MCF‐7, T47D, HCC1937, Bcap37, NHFB, HBL‐100 cell lines	*Homo sapiens*	Target the UFM1/SLC7A11 pathway to induce SLC7A11‐mediated ferroptosis in breast cancer cells	^195^
Metformin	MDA‐MB‐231 cell line	*Homo sapiens*	Target the miR‐324‐3p/GPX4 axis to induce ferroptosis in breast cancer cells	^196^
Metformin	MCF‐7 cell line	*Homo sapiens*	Inhibit lncRNA H19‐induced ferroptosis in breast cancer cells	^197^
Glycyrrhizic acid	MDA‐MB‐231 cell line	*Homo sapiens*	Activates NADPH oxidase and iNOS in cells, promotes the generation of ROS and RNS, leads to reduced GSH and GPX activities and triggers ferroptosis	^210^
Arnicolide D	Breast cancer	*Homo sapiens*	Decreases the expression of GPX4, leading to increased accumulation of Fe^2+^ and MDA, ultimately triggering ferroptosis in breast cancer cells	^213^
Levistilide A	MDA‐MB‐231 and MCF‐7 cell lines	*Homo sapiens*	Activates the Nrf2/HO‐1 signalling pathway and significantly enhances the induction of ROS‐mediated ferroptosis	^217^
Eupaformosanin	MDA‐MB‐231 and MDA‐MB‐468 cell lines	*Homo sapiens*	Induction of ferroptosis in breast cancer cells by ubiquitination of mutated p53	^222^
Polyphyllin III	MDA‐MB‐231 cell line	*Homo sapiens*	Induction of ferroptosis in breast cancer cells via ACSL4	^232^
Cryptotanshinone	MDA‐MB‐231 and 4T1‐Luc cell lines	*Homo sapiens* and *Mus musculus*	Increase intracellular total iron, ferrous iron, ROS, lipid peroxidation process and its product 4‐HNE to induce ferroptosis	^236^
Dihydroisotanshinone I	MDA‐MB‐231 and MCF‐7 cell lines	*Homo sapiens*	Inhibit GPX4 protein expression to induce ferroptosis in breast cancer cells	^96^
Quercetin	MDA‐MB‐231 and MCF‐7 cell lines	*Homo sapiens*	Enhances TFEB expression and nuclear transcription and induces ferroptosis in breast cancer	^254^
Tetrandrine	MDA‐MB‐231 and MCF‐7 cell lines	*Homo sapiens*	Inhibit the GPX4 expression and activate the NCOA4‐mediated ferritophage in breast cancer cells	^263^
Gallic acid	MDA‐MB‐231 cell line	*Homo sapiens*	Increase ROS production, reduce GPX4 activity and increase lipid peroxidation to induce ferroptosis in breast cancer cells	^276^
Artemisinin	MDA‐MB‐231 cell line	*Homo sapiens*	Increase ROS and MDA and decrease GSH and GPX4 to promote ferroptosis of breast cancer cells	^283^
Ungeremine	MDA‐MB‐231 cell line	*Homo sapiens*	Induction of ferroptosis in breast cancer mediated by caspase activation, MMP alterations, and increased ROS production	^284^
Indirubin	4T1 cell line	*Mus musculus*	Reduces GSH levels, increases MDA and 4‐HNE levels, and reduces GPX4, thereby inducing ferroptosis in breast cancer cells	^288^

### 7.1 Curcumin

Curcumin, chemically known as diferuloylmethane, has a long history of safe consumption as a natural food colourant and has shown promising applications in pharmacology and cancer therapy.^176^, ^177^ Derived from the turmeric rhizome (Curcuma longa), curcumin exerts anti‐proliferative and pro‐apoptotic effects in various cancer cell lines, including pancreatic cancer,^178^ prostate cancer^179^ and malignant mesothelioma.^180^ Its ability to remove ROS and activate antioxidant response elements effectively inhibits ROS‐induced lipid peroxidation.^181^ Recent studies have revealed curcumin's connection to ferroptosis through its modulation of heme oxygenase 1 (HO‐1). Upregulation of HO‐1 promotes iron accumulation and ROS production, thereby influencing ferroptosis.^182^ Apart from HO‐1 regulation, curcumin influences multiple molecular targets and signalling pathways. Administration of curcumin formulations with enhanced bioavailability to breast cancer patients resulted in reduced systemic inflammation and improved quality of life.^180^ Li et al.^183^ reported that curcumin induces significant intracellular iron accumulation, ROS generation, lipid peroxidation and malondialdehyde production while decreasing glutathione levels. Curcumin also upregulates multiple ferroptosis target genes, particularly those related to redox regulation, including HO‐1. These findings shed light on the molecular and cellular characteristics of curcumin‐induced ferroptosis in breast cancer cells, where HO‐1 plays a crucial role. In relation to triple‐negative breast cancer, Cao et al.^184^ demonstrated that curcumin dose‐dependently inhibits the viability of MDA‐MB‐453 and MCF‐7 cells. Further investigations revealed that curcumin promotes SLC1A5‐mediated ferroptosis, indicating its potential as a therapeutic agent for breast cancer treatment.

### 7.2 Metformin

Metformin, originally derived from a grass called ‘goat bean’, a legume native to Europe also known as French clove, was found to possess a remarkable curative effect but was initially unsuitable for clinical practice due to its high toxicity. Scientists attempted to modify the chemical structure of goistline (prenguanidine), leading to the synthesis of a series of guanidine derivatives, including metformin in 1922. Metformin quickly passed human studies and was successfully applied clinically, emerging as a potential treatment for conditions beyond diabetes, such as cancer prevention and anti‐ageing effects.^185^, ^186^, ^187^, ^188^, ^189^

The investigation of metformin's anti‐tumour efficacy and underlying mechanisms has become a prominent focus in cancer research. Metformin exerts its anti‐tumour effects through various mechanisms. It can indirectly influence tumorigenesis by reducing insulin levels systemically or directly inducing energy stress.^188^ It has been found that metformin inhibits tumorigenesis by modulating endocrine and metabolic states, resulting in decreased serum glucose and insulin levels. Additionally, metformin directly acts on tumour cells, exerting anti‐tumour effects through AMPK‐dependent and/or AMPK‐independent signalling pathways. Notably, metformin activates AMP‐activated protein kinase (AMPK), deactivates the mammalian target of rapamycin (mTOR), modulates intracellular ROS levels and impairs mitochondrial function.^190^ Metformin's impact on cellular iron homeostasis has also been observed, potentially affecting cellular metabolism.^191^ Despite the gradual elucidation of metformin's regulation of cell death pathways like apoptosis and autophagy, the primary mechanism responsible for metformin‐mediated tumour suppression remains uncertain.^192^, ^193^, ^194^ Recent studies, however, have identified metformin's ability to induce ferroptosis in an AMPK‐independent manner to inhibit tumour growth. Particularly, metformin disrupts the UFMylation process of the vital ferroptosis regulator protein SLC7A11, leading to its destabilization.^195^ Combining metformin with sulfasalazine, a systemic Xc‐ inhibitor, has demonstrated synergistic effects in inducing ferroptosis and inhibiting breast cancer cell proliferation. In triple‐negative breast cancer, Huo et al. discovered that metformin induces ferroptosis in MDA‐MB‐231 cells by upregulating the expression of miR‐324‐3p.^196^ Thus, metformin targets the miR‐324‐3p/GPX4 axis to induce ferroptosis in breast cancer. Another intriguing avenue involves noncoding RNAs, as metformin may induce ferroptosis by inhibiting the autophagy process of lncRNA H19 in breast cancer.^197^

### 7.3 Glycyrrhetinic acid

Glycyrrhetinic acid, also known as 18‐β‐glycyrrhetinic acid, is the primary active compound derived from the medicinal herb Glycyrrhizae.^198^ This compound exhibits a broad spectrum of anticancer activities in various experimental systems, including hepatocellular carcinoma, gastric cancer, breast cancer, lung cancer, leukaemia and thyroid cancer.^199^, ^200^, ^201^, ^202^, ^203^ The cytotoxic and anti‐tumour properties of glycyrrhetinic acid can be attributed to the triterpene functional groups (COOH and OH) present in its molecular structure.^204^

Glycyrrhetinic acid shows promise as a drug delivery vehicle for hepatocellular carcinoma treatment by utilizing receptor‐mediated endocytosis, effectively addressing its low lipophilicity and poor bioavailability.^205^ In an interesting study, 18β‐glycyrrhetinic acid suppressed tumour growth and induced apoptosis by activating the mitochondria‐mediated ROS/mitogen‐activated protein kinase pathway in transplanted nude mice with experimental pituitary adenoma.^206^ Glycyrrhetinic acid derivatives selectively target specific protein (Sp) transcription factors, promoting ROS generation and inhibiting rhabdomyosarcoma growth.^207^ Furthermore, glycyrrhetinic acid has been reported to induce autophagy in MDA‐MB‐231 breast cancer cells by activating the ROS‐mitochondrial pathway, with ferroptosis identified as an autophagic cell death process.^208^, ^209^

A noteworthy discovery by Wen et al.^210^ revealed that glycyrrhetinic acid promotes the generation of ROS and RNS by activating NADPH oxidase and inducible nitric oxide synthase (iNOS) in TNBS cells. This leads to reduced activity of GSH and GPX, aggravation of lipid peroxidation, and initiation of ferroptosis.

### 7.4 Arnicolide D

Arnicolide D, a sesquiterpene lactone derived from Centipedeaceae herbs, has exhibited promising anti‐colorectal and anti‐nasopharyngeal cancer properties, along with inhibiting the NF‐κB, AKT and STAT3 signalling pathways.^211^, ^212^ A recent study discovered that Arnicolide D effectively reduces the expression of GPX4, leading to increased accumulation of Fe^2+^ and MDA, ultimately triggering ferroptosis. Furthermore, Arnicolide D induces oxidative stress, activating multiple cell death pathways, effectively impeding the growth and invasion of breast cancer cells. These findings underscore the significant anti‐tumour potential of Arnicolide D.^213^

### 7.5 Levistilide A

Levistilide A, an active compound derived from Rhizoma Chuanxiong widely used in traditional oriental medicine for cancer treatment, has garnered significant attention. Previous investigations have revealed its potential to inhibit liver fibrosis by reducing the formation of hepatic sinusoidal capillaries.^214^ Additionally, Levistilide A exerts beneficial effects on the proliferation of hepatic stellate cells by regulating cell cycle progression and apoptosis.^215^ Importantly, it has been discovered that Levistilide A promotes apoptosis in colon cancer cells through the induction of endoplasmic reticulum stress, mediated by the accumulation of ROS.^216^ These findings collectively suggest that the administration of Levistilide A may impede tumour development by influencing critical cellular processes involved in ferroptosis. In a recent study, Levistilide A noticeably decreased cell viability and disrupted mitochondrial structure and function in breast cancer cells in a dose‐dependent manner. Furthermore, treatment with Levistilide A significantly enhanced the induction of ROS‐mediated ferroptosis by activating the Nrf2/HO‐1 signalling pathway.^217^

### 7.6 Eupaformosanin

Sesquiterpene lactones have gained significant attention due to their remarkable biological activities, which include anticancer, anti‐inflammatory, and antimalarial properties.^218^, ^219^ Recent studies have revealed that sesquiterpene lactones can induce apoptosis and ferroptosis by targeting the ubiquitination of GPX4 in TNBC.^220^ Among these compounds, Eupaformosanin, a natural product extracted from Eupatorium cannabinum Linn, has exhibited inhibitory effects on the metabolism of Ehrlich ascites cells by suppressing DNA synthesis.^221^ Wei et al.^222^ conducted research demonstrating that Eupaformosanin effectively suppresses the viability of TNBC cells. Notably, Eupaformosanin‐induced cell death is implicated in mitochondrial apoptosis, characterized by the disruption of mitochondrial membrane potential (Δψm) and the accumulation of mitochondrial ROS. The apoptosis inhibitor Z‐VAD mitigates the cell death induced by Eupaformosanin. Interestingly, treatment with Eupaformosanin enhances ferroptosis‐induced cell death, as evidenced by the accumulation of lipid ROS, depletion of GSH, and increased iron levels. The inhibition of ferroptosis by ironstatin‐1 (Fer‐1), deferoxamine (DFO) and lipstatin‐1 (lip‐1) effectively blocks these events, underscoring the role of ferroptosis in promoting Eupaformosanin‐induced cell death. Furthermore, Eupaformosanin regulates the ubiquitination of mutant p53, and its signalling is implicated in Eupaformosanin‐induced apoptosis and ferroptosis, which can be rescued by silencing mutant p53 in TNBC cells. Importantly, Eupaformosanin exhibits anti‐TNBC effects by inducing apoptosis and ferroptosis in vivo. Overall, Eupaformosanin holds promise as a potential therapeutic agent for TNBC, targeting apoptosis and ferroptosis through the ubiquitination of mutant p53.

### 7.7 Polyphyllin III

Polyphyllin III, also known as diosgenin, represents the primary saponin derived from the rhizome of Paris multifoliate.^223^ Its antiproliferative effects have been demonstrated in various cancers, including gastric, hepatocellular, lung and breast cancers.^224^, ^225^, ^226^, ^227^, ^228^ Mechanisms underlying its anticancer activity encompass cell cycle arrest, apoptosis, autophagy, and GSDME‐dependent pyroptosis, as proposed by earlier studies.^227^, ^229^, ^230^, ^231^ In a recent investigation, One et al. elucidated the proliferation‐inhibiting effect of polyphyllin III on MDA‐MB‐231 triple‐negative breast cancer cells, primarily attributed to elevated lipid peroxidation mediated by ACSL4 and subsequent induction of ferroptosis. Notably, the attenuation of chlorophyll III‐induced ferroptosis was observed upon partial loss of ACSL4. Furthermore, treatment with chlorophyll III induced a KLF4‐mediated upregulation of xCT, a negative regulator of ferroptosis, providing a protective effect. Intriguingly, MDA‐MB‐231 cells exhibited sensitivity to polyphyllin III when combined with the xCT inhibitor SAS or upon downregulation of KLF4. Moreover, in an in vivo xenograft model, SAS significantly increased the sensitivity of MDA‐MB‐231 breast cancer cells to chlorophyll III, potentially by enhancing intracellular lipid peroxidation and ferroptosis. Collectively, the findings from this study highlight the ability of polyphyllin III to induce ferroptosis in MDA‐MB‐231 breast cancer cells through ACSL4, underscoring its potential as an effective anticancer agent.^232^

### 7.8 Cryptotanshinone

Cryptotanshinone is a monomer with various pharmacological activities extracted from Salvia miltiorrhiza, which has various effects such as anticancer, anti‐inflammatory, anti‐oxidation, anti‐diabetes and anti‐obesity.^233^ Cryptotanshinone comes from natural medicines and has the advantages of low toxicity and wide indications.^233^, ^234^, ^235^ Yan et al. found that cryptotanshinone inhibits tumour growth by promoting ferroptosis in granulocytic myeloid‐derived suppressor cells and TNBC cells through proteomics, single‐cell transcriptome sequencing, ferroptosis phenotype detection, DARTS, microthermophoresis and molecular docking.^236^

### 7.9 Dihydroisotanshinone I

Dihydroisotanshinone I, a constituent derived from Salvia miltiorrhiza (Danshen), holds a significant place in traditional Chinese herbal medicine due to its extensive clinical applications.^237^, ^238^ Danshen has proven beneficial in improving the survival rates of patients diagnosed with prostate cancer, lung cancer and colon cancer.^239^, ^240^, ^241^ Previous investigations have highlighted the anti‐growth properties of various forms of tanshinone, such as tanshinone IIA, acetyltanshinone IIA and tanshinone I, in breast cancer cell lines through the induction of apoptosis.^242^, ^243^, ^244^, ^245^, ^246^ Recently, a study has discovered that dihydroisotanshinone I effectively inhibits the growth of breast cancer cells, including MCF‐7 and MDA‐MB‐231 cells. Moreover, dihydroisotanshinone I induces both apoptosis and ferroptosis in these breast cancer cells. Additionally, it inhibits the protein expression of GPX4. Notably, in vivo studies utilizing a xenograft nude mouse model have further demonstrated that dihydroisotanshinone I treatment significantly suppresses the final tumour volume without any adverse effects.^96^

### 7.10 Quercetin

Quercetin, a flavonoid abundant in plants, can be found in various fruits and vegetables including onions, grapes, apples and green leafy vegetables.^247^ Its ability to inhibit the proliferation of various tumours has been well documented.^248^ Additionally, quercetin is involved in numerous physiological activities, including antioxidant effects, inflammation inhibition, fibrosis suppression and limitation of viral proliferation.^249^, ^250^ The anti‐tumour effects of quercetin can be attributed to various mechanisms such as cell cycle arrest, autophagy induction and activation of intrinsic and extrinsic apoptotic pathways.^251^, ^252^ Unlike conventional chemotherapy with severe side effects, oral administration of quercetin over several months has been proven safe and effective without significant adverse reactions.^253^ In a recent study, it was demonstrated that quercetin induces cell death in breast cancer cells and concentration‐dependently elevates iron levels, MDA (malondialdehyde) and carbonyl proteins. Moreover, TFEB expression was predominantly observed in the nucleus and low in the cytoplasm. Enhanced TFEB expression promoted the upregulation of the lysosome‐related LAMP‐1 gene, facilitating the degradation of ferritin and subsequent release of ferric ions. Notably, TFEB siRNA or chloroquine effectively blocked the aforementioned pharmacodynamic effects of quercetin. These findings suggest that quercetin can enhance TFEB expression and nuclear transcription, inducing ferroptosis as a pharmacological approach to eliminate breast cancer cells.^254^

### 7.11 Tetrandrine

Tetrandrine, a natural product extensively utilized in China for the treatment of pulmonary fibrosis and arthritis,^255^, ^256^ has been reported to exhibit anti‐tumour activity in chronic myelogenous leukaemia and human laryngeal carcinoma.^257^, ^258^ However, its clinical application remains limited due to its hydrophobic nature and poor solubility in water. A novel tetrahydrogen salt called Tetrahydrogen citrate (TetC), possessing high water solubility, has demonstrated potent anti‐tumor effects in chronic myeloid leukaemia.^259^ Previous studies have elucidated that Tetrandrine inhibits the growth of breast cancer cells by inducing apoptosis and autophagy.^260^, ^261^, ^262^ In a recent study conducted by Yin et al.,^263^ TetC exhibited significant inhibitory effects on the proliferation and migration of MCF7 and MDA‐MB‐231 cells. It was also found to markedly increase the mRNA levels of prostaglandin‐endoperoxide synthase 2 (Ptgs2), a ferroptosis marker. Furthermore, TetC exhibited substantial inhibition of glutathione peroxidase 4 (GPX4) and ferritin heavy chain 1 (FTH1) expression while promoting the expression of nuclear receptor coactivator 4 (NCOA4) in MCF7 and MDA‐MB‐231 cells, even in the presence of erastin or Ras selective lethal 3 (RSL3). Overall, TetC‐induced cell death primarily occurred through the mechanism of ferroptosis. Notably, TetC‐induced ferritin cell death demonstrated inhibition of GPX4 expression and activation of NCOA4‐mediated ferritin phagocytosis in breast cancer cells.

### 7.12 Gallic acid

Phenolic compounds, which are secondary metabolites found in plants, consist of aromatic rings with hydroxyl groups. To date, over 8000 different types of natural phenolic compounds have been identified.^264^, ^265^ Plant‐derived phenolic compounds encompass a diverse group, including flavonoids, ligands, tannins, xanthines and coumarins.^266^, ^267^ These compounds are renowned for their anticancer activity and their ability to combat diseases associated with oxidative stress.^268^, ^269^, ^270^ Among the known polyphenols in nature, gallic acid stands out.^271^, ^272^, ^273^ Also known as 3,4,5‐trihydroxybenzoic acid, gallic acid boasts important anticancer properties with antioxidant effects.^274^, ^275^ In a study conducted by Khorsandi et al.,^276^ it was observed that low‐level laser irradiation alone does not induce cell death in normal human cells or cancer cells. However, when gallic acid treatment followed the irradiation, cell viability in cancer cells decreased significantly compared to treatment with gallic acid alone, while the viability of normal human cells remained relatively unaffected.

### 7.13 Artemisinin

Artemisinin, a sesquiterpene lactone containing a peroxide group,^277^, ^278^ was isolated from the traditional Chinese plant Artemisia annua and exhibited desirable anti‐tumour activity in various cancer cell lines.^279^, ^280^ Accumulating evidence indicates that cancer cells contain more intracellular iron pools than normal cells and that iron‐mediated cleavage of peroxide bridges allows artemisinins to selectively cause cell death in a variety of cancer cell lines.^279^, ^281^ Iron‐dependent anti‐tumour activity has attracted increasing attention in ART‐mediated ferroptosis.^279^ Mechanistically, artemisinin can induce lysosomal degradation of ferritin, independent of autophagy, increase cellular levels of ferrous ions and sensitize cells to ferroptosis.^282^ A recent study shows that artemisinin and its derivatives have been investigated as potential anticancer agents for the treatment of highly aggressive breast cancer by inducing ferroptosis through iron‐mediated cleavage of peroxide bridges.^283^

### 7.14 Ungeremine

Many naturally occurring alkaloids and their derivatives have excellent cytotoxic activity, such as vinblastine and vincristine, and have been clinically established anticancer drugs for decades. The latest study shows that Ungeremine exhibits cytotoxic activity against nine cancer cell lines tested, including drug‐sensitive and MDR phenotypes. Ungeremine can induce ferroptosis, necroptosis, autophagy and apoptosis mediated by caspase activation, MMP alteration and increased ROS production.^284^

### 7.15 Indirubin

Indirubin, a bisindole compound, serves as the active ingredient in the traditional Chinese medicine Qingdai. The indigotin and its derivatives found in indirubin exhibit notable anticancer effects by modulating various signalling pathways such as PI3K/AKT/mTOR, NF‐κB, MAPK, JAK/STAT3 and more, thereby regulating the expression of cyclin‐dependent kinases (CDK), GSK‐3β, Bax, Bcl‐3, C‐MYC, matrix metalloproteinases (MMP) and focal adhesion kinase (FAK).^285^, ^286^, ^287^ In a study conducted by Kuang,^288^ it was discovered that indirubin stimulates lipid peroxidation in breast cancer cells, resulting in reduced glutathione (GSH) levels, increased malondialdehyde (MDA) and 4‐Hydroxynonenal (4‐HNE) levels and decreased glutathione peroxidase 4 (GPX4) expression. These changes ultimately induce ferroptosis in breast cancer cells. The underlying mechanism seems to involve the promotion of GSK‐3β phosphorylation at the Ser9 site and upregulation of PTGS2, which collectively foster ferroptosis and confer resistance against breast cancer.

## 8 PROSPECT

A recent study briefly outlined the regulatory effects of phytochemicals on ferroptosis in breast cancer, but the detailed mechanisms of ferroptosis in breast cancer were not extensively explored. This current research aims to further enrich our understanding of the various natural product‐mediated mechanisms regulating ferroptosis and expand our knowledge of the regulatory mechanisms involving ferroptosis‐related signalling pathways and associated metabolic pathways.^289^ These signalling pathways hold great promise as potential targets for anti‐breast cancer drugs. Furthermore, there is an urgent need to explore the intricate mechanisms underlying this novel form of cell death in breast cancer, particularly within the context of lipid peroxidation and ferroptosis‐sensitive metabolic pathways. Key areas of investigation include thiol metabolism (including selenium), fatty acid metabolism, iron regulation, the mevalonate pathway and mitochondrial respiration. This comprehensive review highlights the discovery of ferroptosis in breast cancer patients and the utilization of relevant in vivo and in vitro models. It becomes evident that advancing our knowledge regarding ferroptosis in the pathological mechanisms of breast cancer is crucial. By unravelling this process, we can potentially identify targets for breast cancer treatment and shed light on additional genes and mechanisms that regulate ferroptosis.

In addition, it is crucial to note that ferroptosis is not limited solely to breast cancer tissue but can also occur in normal tissue. A recent study reported the identification of the luminal androgen receptor (LAR) subtype in TNBC, characterized by an upregulation of oxidative phospholipid ethanolamines and glutathione metabolism, particularly GPX4. This upregulation allows the induction of ferroptosis through GPX4 inhibition. Furthermore, this discovery demonstrates that inhibiting GPX4 not only induces tumour ferroptosis but also enhances anti‐tumour immunity. Combination therapy of GPX4 inhibitors with anti‐PD1 treatment shows superior efficacy compared to monotherapy. Clinically, higher GPX4 expression in the immune therapy cohort correlates with lower cytolytic activity scores and poorer prognosis.^290^ Therefore, it is crucial for potential future ferroptosis treatments to consider the possibility of serious complications. This necessitates more comprehensive studies on the underlying mechanisms of ferroptosis in breast cancer, with a focus on identifying additional factors involved in its regulation and suitable biomarkers for breast cancer patients. Several current factors implicated in ferroptosis, namely FSP1, GPX4, ACSL4, SLC7A11, ALOXs, and GCH1, show promise as potential markers. High expression levels of these markers could potentially indicate that patients are more responsive to therapeutic drugs employing ferroptosis as a treatment strategy.

In addition to the aforementioned pathological mechanisms of ferroptosis in breast cancer, it is crucial to consider the potential utility of ferroptosis as a monitoring tool for traditional and biologically targeted drugs in breast cancer intervention. While some studies have indicated that iron and lipid peroxidation levels can serve as biomarkers for monitoring the efficacy of anticancer drugs, further exploration and validation of these markers through future clinical trials are warranted. Furthermore, given the complex nature of breast cancer, various forms of programmed cell death, such as autophagy, pyroptosis, and apoptosis, occur simultaneously, forming an intricate network of interactions with ferroptosis. Determining which form of programmed cell death predominates and exploring the potential synergistic effects of combined therapies, such as inhibitors targeting specific cell signalling pathways, requires extensive research both in the laboratory and clinical settings. It is particularly important to consider the clinical heterogeneity across different types of breast cancer, acknowledging that current data primarily stem from cellular and animal models. Consequently, a longer clinical path lies ahead to address the aforementioned challenges.

Regarding natural plant active ingredients, current research suggests that several classes of compounds, such as flavonoids, alkaloids, phenols, lignans and saponins, have shown the potential to inducing ferroptosis in breast cancer. However, most of these findings are based on preclinical experiments, lacking extensive large‐scale and long‐term clinical research verification. Therefore, further clinical trials are necessary to validate their effectiveness in the future. By comprehensively examining natural compounds, it is anticipated that novel lead compounds capable of inducing ferroptosis in breast cancer can be developed, thereby paving the way for the development of new inducers specifically targeting tumour ferroptosis.

Last, the comprehensive management strategy for breast cancer patients is a promising direction for future research. Clinical trials should focus on how patients can obtain optimal treatment plans through therapeutic strategies targeting ferroptosis while mitigating complications. This may involve investigating the combination of therapies that induce ferroptosis with immune checkpoint inhibitors and other treatment strategies, thus forming an innovative combination strategy for immunotherapy. In conclusion, it is expected that targeting the mechanism of ferroptosis in breast cancer will provide a new direction for the comprehensive treatment of breast cancer in the future.

## AUTHOR CONTRIBUTIONS

**Anqi Ge:** Conceptualization (equal); data curation (equal); formal analysis (equal); funding acquisition (equal); writing – original draft (equal). **Qi He:** Data curation (equal); formal analysis (equal); funding acquisition (equal); investigation (equal); writing – original draft (equal). **Da Zhao:** Conceptualization (equal); data curation (equal); formal analysis (equal); writing – original draft (equal). **Yuwei Li:** Writing – review and editing (equal). **Junpeng Chen:** Writing – review and editing (equal). **Ying Deng:** Writing – review and editing (equal). **Wang Xiang:** Data curation (equal); formal analysis (equal); funding acquisition (equal); writing – original draft (equal). **Hongqiao Fan:** Data curation (equal); formal analysis (equal); funding acquisition (equal); investigation (equal). **Shiting Wu:** Data curation (equal); formal analysis (equal); funding acquisition (equal); investigation (equal). **Lifang Liu:** Conceptualization (equal); data curation (equal); formal analysis (equal); funding acquisition (equal); writing – original draft (equal). **Yue Wang:** Data curation (equal); formal analysis (equal); funding acquisition (equal); methodology (equal). **Yan Li:** Formal analysis (equal); funding acquisition (equal); investigation (equal).

## FUNDING INFORMATION

This work was supported by the Scientific Research Fund of Hunan Provincial Education Department (no. 22B0364), the Hunan Provincial Natural Science Foundation of China (no. 2021JJ40421).

## CONFLICT OF INTEREST STATEMENT

We declare no competing interests.

## Data Availability

Data sharing not applicable to this article as no datasets were generated or analysed during the current study.
